# Use of Gastrointestinal Anastomosis Stapler for Harvest of Gracilis Muscle and Securing It in the Face for Facial Reanimation: A Novel Technique

**Published:** 2010-04-08

**Authors:** Sachin M. Shridharani, Sahael M. Stapleton, Richard J. Redett, Michael Magarakis, Gedge D. Rosson

**Affiliations:** Department of Surgery, Division of Plastic, Reconstructive and Maxillofacial Surgery, The Johns Hopkins University School of Medicine, Baltimore, Md

## Abstract

**Background:** The primary objective of this study is to report a novel technique that uses the gastrointestinal anastomosis (GIA) stapler for harvesting and securing the gracilis muscle in facial reanimation surgery. **Methods:** We conducted a retrospective chart review with 18 consecutive patients who underwent gracilis muscle flap transfer with or without the use of a GIA stapler. **Results:** Of 11 operations with the GIA stapler, one patient developed a hematoma (donor site) and another required drainage of an abscess (recipient site). Of 8 operations without the use of the stapler, one patient had total flap failure and three required drainage of an abscess (2 recipient sites and 1 donor site). These differences trended toward improvement but were not statistically different. **Conclusions:** The use of the GIA stapler is a fast, safe technique. Larger studies are, however, warranted to further examine this novel approach in order to test precisely what factors of increased efficiency occur, the amount of suture pull-through, and overall tension capable of being applied to the secured staple line.

Microneurovascular gracilis muscle free flap is a well-described muscle transfer in facial paralysis patients.^1^ Good results have been obtained secondary to its ability to be a neurotized muscle flap, affording patients the ability to reanimate facial structures.^1^,^2^ The technique for harvesting has been well described but can have complications^1^,^3^: donor site hematoma, infection, recipient site muscle avulsion, and need for reanchoring.^4^^-^7 Imbricating sutures are often used to reinforce the muscle on the nontendinous side to provide an area to which the facial anchoring sutures are attached to native insertion points on the facial skeleton and muscles.^8^ Although this method has been used for years, we propose a technique that has not been described in the literature.

We have started using the gastrointestinal anastomosis (GIA) stapler (Tyco, Autosuture 3.8-mm thickness, Mansfield, Mass) during the harvest portion of the operation (Fig 1). In addition to increased time efficiency (technique takes 10–15 seconds), surgeons felt that the stapler provided superior hemostasis and stronger points to which sutures could be anchored and attached to facial regions of interest for reanimation.

The primary objective of the study is to report the use of a fast, safe technique for harvesting and securing the gracilis muscle when utilized in facial reanimation surgery. Two groups of patients were compared: those who had undergone gracilis muscle harvest with the traditional technique employing suturing of the proximal and distal ends versus those patients who had undergone gracilis muscle harvest with the use of a GIA stapler. Thus, we aim to elucidate a new technique that has not been previously described. We hypothesized that the new technique described would not have higher complication rates or negatively impact the return of function in the face.

## METHODS

We performed a retrospective review of 18 consecutive patients who had undergone facial reanimation with 19 gracilis free-muscle transfers after facial paralysis over the past 5 years (January 2004 through December 2008). The Johns Hopkins Medicine Institutional Review Board approved this study. The patients ranged in age from 5 to 69 years (mean age = 35.6 years) and were divided into 2 groups (GIA stapler group vs control group).

Demographic data were collected for all 18 patients from their electronic patient records. All preoperative and postoperative facial animations were documented and photographed. We also recorded the surgical technique used in each operation including the method of gracilis muscle harvest. In the control group, the muscle was divided with electrocautery and then suture ligated using 2-O Polysorb (US Surgical, Syneture, Covidien, NY) sutures; study group muscle was harvested using the GIA stapler.

Postoperatively, all patients were evaluated for return of facial animation and potential complications. Complications were defined as flap failure and donor/recipient site hematoma, abscess formation, or postoperative cellulitis requiring antibiotic therapy. Flap failure was considered a “major” complication.

Descriptive statistics (medians, means, frequencies) were calculated to summarize patient and surgery characteristics. Chi-square statistic or Wilcoxson rank-sum test was used when the characteristic or outcome was categorical; the Student *t* test was used when the variable of interest was continuous. All testing was 2-sided at the alpha level of .05.

### Operative procedure for harvesting, extracting, and securing the gracilis muscle

**Figure 1:**
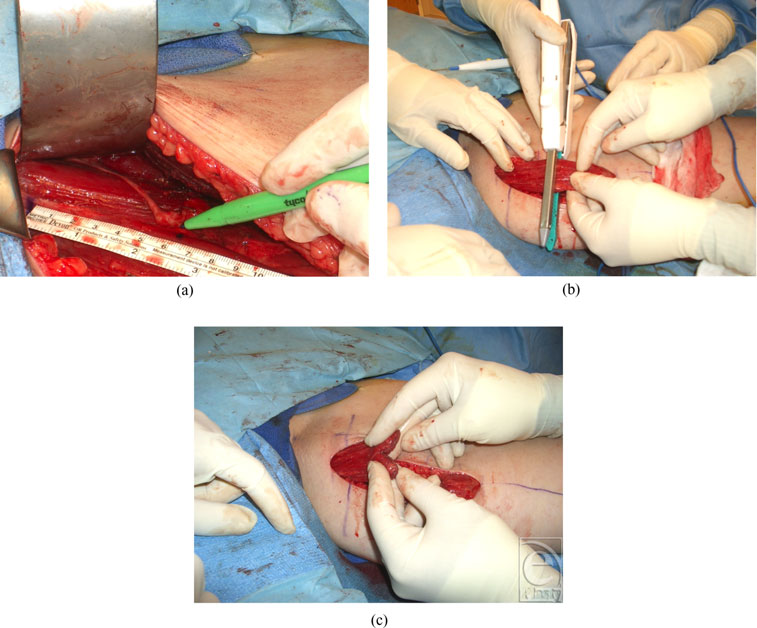
(A) Measurements are made and marked on the gracilis muscle prior to detaching the muscle in the thigh; (B) Intra-operative view of GIA stapler to harvest the gracilis muscle; and (C) Demonstration of how the GIA staple line is easily molded into the shape of the oral commissure.

After the medial thigh is marked, prepped, and draped, incision is made in the medial thigh, posterior to the abductor longus tendon. Fascia is incised to allow for identification of the gracilis muscle. The muscle is freed, and primary and secondary vascular pedicles are identified. The secondary pedicle is clipped and divided; primary pedicle course is followed toward the adductor longus muscle, exposing the branch of the obturator nerve that supplies the gracilis muscle. Approximately 13 cm of the gracilis muscle is marked at 1-cm increments, predetermined by measuring from modiolus to zygomatic arch. Hemoclips are placed at those increments to ensure optimal length during facial inset. One can expect the muscle to recoil after division; hemoclips allow for reference points at the time of inset. After dissecting toward the profunda vessels and obturator foramen, the surgeon ensures adequate vessel and nerve length and then the artery, vein, and nerve of interest are clipped and divided. The muscle is divided at the distal end by placing the muscle between the 2 blades of the GIA stapler and firing (Fig 1). Typically, 60% of the muscle diameter is harvested.

The gracilis flap is transferred to the face. The muscle is inset into the created pocket. The proximal (fascial) aspect of the gracilis muscle is attached into the lateral aspect of the zygomatic arch with interrupted figure-of-8 3-0 Ti-Cron (Syneture, US Surgical Covidien, Mansfield, Mass) sutures. The muscle is positioned using the hemoclips; the medial portion is sutured to the previously placed anchoring sutures. Under microscope guidance, the flap vessels are anastomosed to the facial vessels or the superficial temporal vessels. The motor nerve branch of the gracilis muscle is coapted to the cross-face nerve grafts or masseter motor branch with Tisseel fibrin sealant (Baxter, Deerfield, Ill).

## RESULTS

Ten patients (6 males and 4 females) underwent gracilis muscle harvest, using a GIA stapler, whereas 8 patients (2 males and 6 females) underwent gracilis muscle harvest with the usual approach. Postoperative hospital stay for the GIA stapler and non-GIA stapler group ranged from 1 to 7 days (mean = 3.9 days) and from 2 to 5 days (mean = 3.6 days), respectively. The rate of complications in the GIA stapler group was 18% compared with 50% in the non-GIA stapler group (*P* = .32). Only 1 patient had a “major” complication of flap failure (non-GIA stapler group). Although there were fewer complications in the GIA stapler group, the sample size was not large enough to reflect statistically significant difference in overall complications (Table 1). There was no statistically significant difference between the GIA stapler and traditional groups with respect to return of function after successful muscle transfer (Table 2).

## DISCUSSION

The neurotized gracilis free muscle flap is a versatile muscle flap used with great success in managing patients with facial paralysis.^1^,^4^,^9^ With continued improvement in surgical instruments, surgeons can find useful applications for those tools and use them to advance other surgical techniques.^10^

In a series of 23 adult free-muscle transfers to the face, Terzis and Olivares^11^ found a 13% overall complication rate: venous thrombosis, low function of transferred muscle, and paraesthesia of the lower leg. In a different series of 31 cases of children with facial paralysis, a 6.2% complication rate (hematomas and free-muscle contractures) was found.^7^ Both studies used cross-facial nerve grafting and free-muscle transfer techniques. Chuang^12^ reported the experience with free gracilis transfer for facial reanimation in 249 cases. They categorize their complications in 2 groups: acute (bleeding, saliva leak, flap failure) versus late (lip contracture, widening, asymmetry lagophthalmos, and insufficient muscle mobility). They report an acute complication rate of 2.4% (6 cases); however, up to 30% of their patients presented with late complications, predominantly upper lip contracture. Finally, Terzis and Noah,^3^ in their series of 100 free muscle transfers (gracilis and pectoralis minor in 63 cases and 34 cases, respectively) for facial reanimation, report an intraoperative complication rate of 11%. The authors did not further analyze what the complication rate was within each group (gracilis vs pectoralis minor). They also report 4 cases of postoperative hematomas. There were 4 muscle transplantation failures (4%), three of which occurred in the pectoralis group and one in the gracilis series. We report a 50% overall complication rate for the traditional approach group versus 18% when using the novel GIA stapler approach (only 1 patient had a “major” complication of flap failure). Our complication rate is likely higher because we included cellulitis as a parameter, whereas the other studies cited earlier did not.

Although free-muscle transfer is a useful approach for facial reanimation, its overall functionality has been debated in terms of volume preservation and function recovery of the transplanted muscle over time.^11^,^13^^-^15 We do not report any progressive functional impairment of transplanted muscle over time in our follow-up periods.

We observed a higher rate of overall complications in the traditional approach; however, interpretation of these results should be made cautiously because of the insufficient number of patients who could not support statistically significant differences in complication rates between the 2 groups (*P* = .32). In essence, these complication rates are *not* different. Most or all of the complications reported are unlikely due to the device but more likely surgeon dependent. Also, it is highly unlikely that the GIA stapler could have improved the return of gracilis muscle function, other than the obvious advantage of anchoring the edges of the flap to the modiolus and/or possible improved hemostasis at the donor site. There may be differences in complication rates depending on the seniority and experience of the surgical team; however, we felt that the use of GIA stapler was more efficient when compared with the traditional approaches.

Another significant limitation of our study was the inability to precisely document the potential time savings of this step. In our hands, the use of the GIA stapler takes a matter of seconds. However, Bovie cautery plus oversewing with Vicryl takes us approximately 5 to 10 minutes, but these times were not explicitly documented in the Operative Notes and therefore could not be included in the data of this retrospective chart review. If we were to repeat this study in a prospective manner, these times certainly would be recorded. It is interesting to note that the time savings may not translate into direct cost savings. The approximate cost of a GIA stapler is $700. Assuming that each minute of operating room time costs approximately $20, then it would need to save 35 minutes in order to translate into absolute cost savings. Thus, the benefits would be determined only by indirect monetary considerations such as decreased complication rates or added strength of the suture line at the modiolus, highlighting the need for further study.

This retrospective patient chart review is innovative in that it elucidates the use of a GIA stapler to harvest the gracilis muscle. In our study, we were able to show a new technique of securing the muscle that is efficient and stable and provides an alternative to the traditional form of securing the gracilis muscle. The operating surgeons consistently felt that using the GIA stapler reduced operating time for this specific step and gave a solid anchor compared to the older technique of employing electrocautery and suture ligation for anchoring support.

The stability and safety exemplified in our first 11 patients lend way to future applications of the GIA stapler and need for further studies. Prospective studies should be conducted to test the efficacy of the stapler for other flaps. In addition, prospective, controlled studies should be conducted to test potential operative time reduction when using the GIA stapler versus conventional methods, potential improvements in complication rates, and potential tensile strength the staple line can tolerate versus conventional suture ligation.

## Figures and Tables

**Table 1:** Gracilis muscle transfer complications

Complications	No GIA^a^ stapler (*n* = 8)	GIA stapler (*n* = 11)
Flap failure	1	0
Hematoma	0	1
Recipient site abscess	2	1
Donor site cellulitis	1	0
Total	4 (50%)	2 (18%)

^a^GIA indicates gastrointestinal anastomosis.

**Table 2:** Return of function after successful muscle transfer

Return of function	No GIA^a^ stapler (*n* = 8)	GIA stapler (*n* = 11)
Yes	6	9
No	1	2
Lost to follow-up	1	0
Total (%)	6 (75%)	9 (82%)

^a^GIA indicates gastrointestinal anastomosis.
